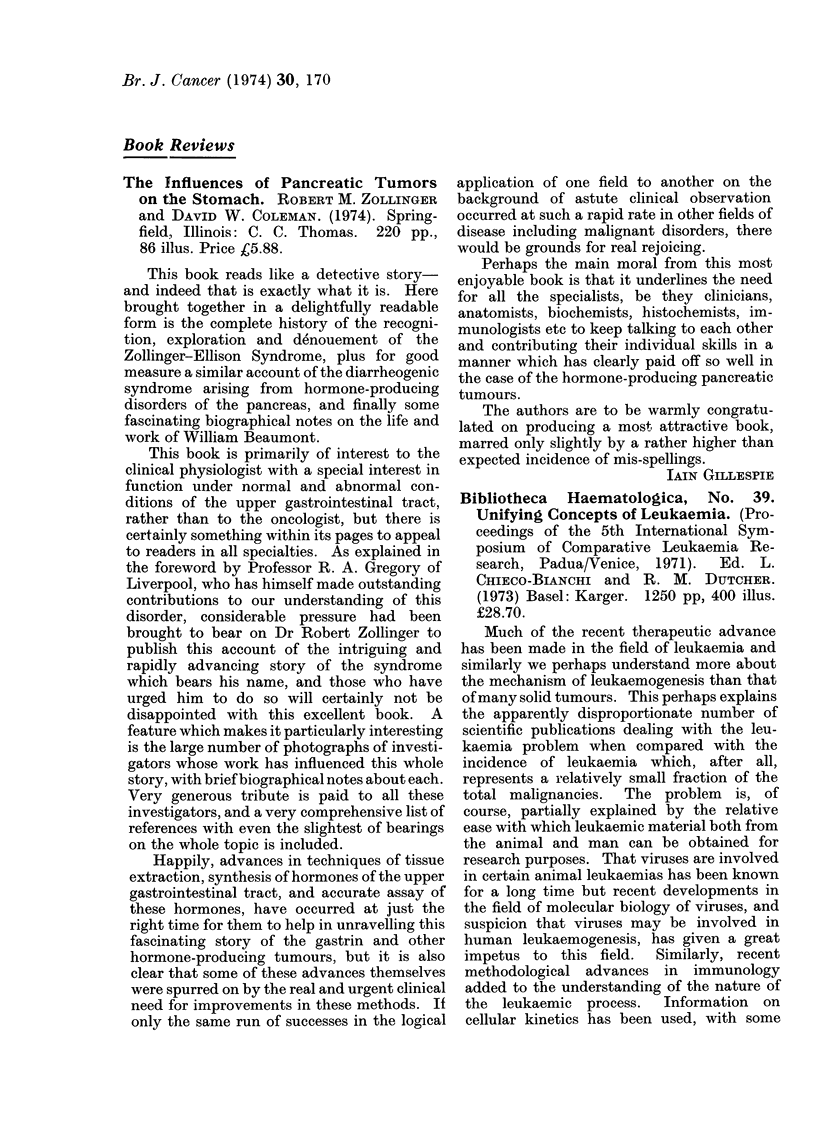# The Influences of Pancreatic Tumors on the Stomach

**Published:** 1974-08

**Authors:** Iain Gillespie


*Br. J. Cancer* (1974) **30**, 170

### *Book Reviews*

**The Influences of Pancreatic Tumors on the Stomach.** ROBERT M. ZOLLINGER and DAVID W. COLEMAN. (1974). Springfield, Illinois: C. C. Thomas. 220 pp., 86 illus. Price £5.88.

This book reads like a detective story—and indeed that is exactly what it is. Here brought together in a delightfully readable form is the complete history of the recognition, exploration and dénouement of the Zollinger–Ellison Syndrome, plus for good measure a similar account of the diarrheogenic syndrome arising from hormone-producing disorders of the pancreas, and finally some fascinating biographical notes on the life and work of William Beaumont.

This book is primarily of interest to the clinical physiologist with a special interest in function under normal and abnormal conditions of the upper gastrointestinal tract, rather than to the oncologist, but there is certainly something within its pages to appeal to readers in all specialties. As explained in the foreword by Professor R. A. Gregory of Liverpool, who has himself made outstanding contributions to our understanding of this disorder, considerable pressure had been brought to bear on Dr Robert Zollinger to publish this account of the intriguing and rapidly advancing story of the syndrome which bears his name, and those who have urged him to do so will certainly not be disappointed with this excellent book. A feature which makes it particularly interesting is the large number of photographs of investigators whose work has influenced this whole story, with brief biographical notes about each. Very generous tribute is paid to all these investigators, and a very comprehensive list of references with even the slightest of bearings on the whole topic is included.

Happily, advances in techniques of tissue extraction, synthesis of hormones of the upper gastrointestinal tract, and accurate assay of these hormones, have occurred at just the right time for them to help in unravelling this fascinating story of the gastrin and other hormone-producing tumours, but it is also clear that some of these advances themselves were spurred on by the real and urgent clinical need for improvements in these methods. If only the same run of successes in the logical application of one field to another on the background of astute clinical observation occurred at such a rapid rate in other fields of disease including malignant disorders, there would be grounds for real rejoicing.

Perhaps the main moral from this most enjoyable book is that it underlines the need for all the specialists, be they clinicians, anatomists, biochemists, histochemists, immunologists etc to keep talking to each other and contributing their individual skills in a manner which has clearly paid off so well in the case of the hormone-producing pancreatic tumours.

The authors are to be warmly congratulated on producing a most attractive book, marred only slightly by a rather higher than expected incidence of mis-spellings.

IAIN GILLESPIE